# Relevance of Frequency-Domain Analyses to Relate Shoe Cushioning, Ground Impact Forces and Running Injury Risk: A Secondary Analysis of a Randomized Trial With 800+ Recreational Runners

**DOI:** 10.3389/fspor.2021.744658

**Published:** 2021-11-11

**Authors:** Laurent Malisoux, Paul Gette, Anne Backes, Nicolas Delattre, Jan Cabri, Daniel Theisen

**Affiliations:** ^1^Department of Population Health, Physical Activity, Sport and Health Research Group, Luxembourg Institute of Health, Luxembourg, Luxembourg; ^2^Department of Population Health, Human Motion, Orthopedics, Sports Medicine and Digital Methods Research Group, Luxembourg Institute of Health, Luxembourg, Luxembourg; ^3^Decathlon Sports Lab, Movement Sciences Department, Decathlon SA, Villeneuve d'Ascq, France; ^4^Luxembourg Institute of Research in Orthopedics, Sports Medicine and Science, Luxembourg, Luxembourg; ^5^ALAN–Maladies Rares Luxembourg, Luxembourg, Luxembourg

**Keywords:** footwear, biomechanics, injury prevention, kinetics, frequency-domain analysis, prospective study

## Abstract

Cushioning systems in running shoes are used assuming that ground impact forces relate to injury risk and that cushioning materials reduce these impact forces. In our recent trial, the more cushioned shoe version was associated with lower injury risk. However, vertical impact peak force was higher in participants with the Soft shoe version. The primary objective of this study was to investigate the effect of shoe cushioning on the time, magnitude and frequency characteristics of peak forces using frequency-domain analysis by comparing the two study groups from our recent trial (Hard and Soft shoe group, respectively). The secondary objective was to investigate if force characteristics are prospectively associated with the risk of running-related injury. This is a secondary analysis of a double-blinded randomized trial on shoe cushioning with a biomechanical running analysis at baseline and a 6-month follow-up on running exposure and injury. Participants (*n* = 848) were tested on an instrumented treadmill at their preferred running speed in their randomly allocated shoe condition. The vertical ground reaction force signal for each stance phase was decomposed into the frequency domain using the discrete Fourier transform. Both components were recomposed into the time domain using the inverse Fourier transform. An analysis of variance was used to compare force characteristics between the two study groups. Cox regression analysis was used to investigate the association between force characteristics and injury risk. Participants using the Soft shoes displayed lower impact peak force (*p* < 0.001, *d* = 0.23), longer time to peak force (*p* < 0.001, *d* = 0.25), and lower average loading rate (*p* < 0.001, *d* = 0.18) of the high frequency signal compared to those using the Hard shoes. Participants with low average and instantaneous loading rate of the high frequency signal had lower injury risk [Sub hazard rate ratio (SHR) = 0.49 and 0.55; 95% Confidence Interval (CI) = 0.25–0.97 and 0.30–0.99, respectively], and those with early occurrence of impact peak force (high frequency signal) had greater injury risk (SHR = 1.60; 95% CI = 1.05–2.53). Our findings may explain the protective effect of the Soft shoe version previously observed. The present study also demonstrates that frequency-domain analyses may provide clinically relevant impact force characteristics.

**Clinical Trial Registration:** https://clinicaltrials.gov/, identifier: 9NCT03115437.

## Introduction

Running is characterized by the repetition of many, almost identical movements with limited variations (Mann et al., 2015). During the stance phase of each step, a ground reaction force (GRF) is applied to the body. The shape of the vertical component of the GRF over time is approximately that of a mass-spring impact (McMahon and Cheng, 1990). In most runners, two distinct peaks are easily detectable on a plot of the vertical GRF vs. time (Malisoux et al., 2021). The first peak (Fz1, often termed vertical impact peak force) occurs within the first 50 ms after initial contact and is referred to as the impact peak, while the second peak (Fz2) occurring approximately at mid stance phase is referred to as the active peak. Fz1 originates from the rapid deceleration of the support leg segments at initial contact, although the magnitude of the peak mainly depends on the contribution of the rest of the body. Fz2 is associated with the active motion of the rest of the body when the center of mass reaches its minimum vertical position during the stance phase (Bobbert et al., 1991). While Fz1 may not always be visually detectable in time-domain plots in runners with specific foot strike patterns (i.e., mid-and forefoot strike), (Lieberman et al., 2010), some studies provided evidence of a vertical impact force in all strike patterns (Gruber et al., 2011, 2017).

The cumulative load applied to the musculoskeletal system resulting from several thousand steps taken during each training session may lead to an overuse injury, especially if inadequate recovery is provided between stress applications (Hreljac, 2004). For decades, it has been assumed that some running-related injuries are typically associated with the landing phase (e.g., stress fracture, tendinopathy), because of the stress resulting from the collision between the body and the ground (Cavanagh and Lafortune, 1980; Bobbert et al., 1991; Wit et al., 1995). The main arguments are that forces observed during distance running are 1.5–2 times larger than those occurring in walking, a rapid increase in force immediately follows initial contact, and a runner covering 20 km per week will cumulate more than 10,000 impacts on each leg over a seven-day period. During the landing phase, eccentric contractions are observed in some of the muscles controlling the movement (e.g., ankle-dorsiflexors or plantar flexors depending on the foot strike pattern, quadriceps muscles), which may cause large forces and high internal mechanical stress (Bobbert et al., 1991). Large muscular forces may also cause high stress on the tendons and their bony attachments. Consequently, biomechanical factors related to vertical GRF have often been studied during running, with Fz1, Fz2 and loading rate being among the most investigated variables.

Surprisingly, the current knowledge on the relationship between impact force characteristics, such as Fz1 or vertical instantaneous loading rate, and injury risk is still inconsistent (Nigg et al., 2015; Theisen et al., 2016; Ceyssens et al., 2019). This is mainly due to small sample sizes, as well as differences in the populations investigated and study designs in research conducted so far (Nigg et al., 2015; Theisen et al., 2016; Ceyssens et al., 2019). Grimston et al. (1991) already observed a greater Fz1 in female runners with a history of stress fracture compared to non-stress-fracture runners, although the question whether these greater forces are a cause or a consequence of having suffered a stress fracture could not be addressed with such a study design. 25 years later, a meta-analysis confirmed that loading rate was higher in runners with a history of stress fracture (van der Worp et al., 2016). A similar effect was found from the studies that included runners with all running-related injury types compared with those without injuries. However, these conclusions are mostly based on case-control designs. A prospective study investigated impact forces as a potential injury risk factor in female runners, but no difference was observed between injured and uninjured runners (Davis et al., 2016). Secondary analyses showed that Fz1 and loading rates were greater in runners with a medically diagnosed injury compared to those who had never reported any previous injury. However, these results were based on a limited sample size (*n* = 32), the analysis was retrospective in nature, and running exposure (i.e., distance or hours of practice) was not taken into account, which severely limits the conclusions that can be drawn. Another plausible explanation for the inconsistent evidence could be that the relationship between impact forces and injury risk, if it exists, is not linear, but U-shaped. In this case, risk factors should not be analyzed as continuous variables, as in most previous studies, but instead be categorized using certain cutoffs (Bahr and Holme, 2003), thus transforming a continuous variable into a categorical or grouping variable. According to this method, runners with values in the upper or lower range of a given variable would be compared to a reference group, e.g., those with values within one standard deviation (SD) around the mean of the entire cohort.

Despite the absence of prospective evidence on the role of impact force in injury development, it has been suggested that paradigms leading to a decrease or elimination of impact forces, such as changes in strike pattern (Lieberman et al., 2010; Cheung and Davis, 2011; Daoud et al., 2012) or increased shoe cushioning (Richards et al., 2009) would reduce injury risk. For instance, a 2-week gait retraining program aiming at lowering loading rate was effective in reducing injury risk in novice runners (Chan et al., 2018a). The protective effect of greater shoe cushioning has also been recently demonstrated in a randomized trial including 800+ recreational runners (Malisoux et al., 2020), where the participants having received the Soft shoe version had a lower injury risk compared to those having received the Hard version (Sub hazard rate ratio–SHR = 1.52; 95% Confidence Interval−95% CI = 1.07 to 2.16; Soft shoe group is the reference), (Malisoux and Theisen, 2020).

In the same trial (Malisoux et al., 2020), all runners were tested in the allocated study shoes at baseline on an instrumented treadmill. One of the main findings was the greater Fz1 observed in the Soft shoe version (Malisoux et al., 2021). This may appear counterintuitive with regards to the initial goal of the cushioning systems (i.e., lower Fz1), but the observation was consistent with previous studies (Baltich et al., 2015; Chan et al., 2018b; Kulmala et al., 2018). An explanation for this “impact peak anomaly” has previously been provided (Shorten and Mientjes, 2011). The vertical GRF signal is actually a superimposition of low frequency (non-impact) and high-frequency (impact) load components (Shorten and Mientjes, 2011). In other words, the true impact force that originates from the collision of the lower leg segment with the ground is superimposed to the active force which depends primarily on the rest of the body (Bobbert et al., 1991). A previous study showed that softer shoes tend not only to attenuate the magnitude of the high-frequency impact peak, but also to delay its occurrence (Shorten and Mientjes, 2011). Consequently, the higher Fz1 observed in shoes with greater cushioning results from the greater relative contribution of the low-frequency load component to Fz1. Furthermore, Fz1 and loading rate were shown to be poorly correlated with tibial load bone metrics computed using a rigid body model (Matijevich et al., 2019), which suggests that Fz1 and loading rate may be unreliable indicators of musculoskeletal loading. Thus, the magnitude of Fz1 is not an appropriate indicator of impact intensity and running shoe impact alteration (Shorten and Mientjes, 2011; Matijevich et al., 2019; Malisoux et al., 2021).

The main purpose of this study was to investigate whether the protective effect of the Soft shoe version observed previously (Malisoux et al., 2020) could be explained by an alteration of decomposed impact force characteristics using frequency-domain analyses. Therefore, the first objective of the study was to investigate the effect of shoe cushioning on the time, magnitude and frequency characteristics of peak forces using frequency-domain analyses by comparing baseline data from the two study groups (i.e., participants who received the Hard and Soft shoe version, respectively). We hypothesized that the Soft shoe version would be associated with a lower impact peak force, a delayed occurrence, and a lower vertical loading rate of the high-frequency signal (Shorten and Mientjes, 2011). Consistently, we also hypothesized that frequency of peak signal power, mean frequency, as well as power sum of the high frequency signal would be lower with the Soft shoe version. However, we did not expect any difference between the experimental groups in the magnitude and timing of the peak force of the low frequency signal. Our secondary objective was to investigate which of these force characteristics measured at baseline are prospectively associated with the risk of incurring a running-related injury. We hypothesized that a lower impact peak force, a delayed occurrence, and a lower vertical loading rate of the high-frequency signal would be associated with lower injury risk. We also expected that lower frequency of peak signal power, mean frequency, as well as power sum of the high frequency signal would be associated with lower injury risk.

## Materials and Methods

### Study Design

The present study is a secondary analysis of a participant-and assessor-blinded randomized trial (ClinicalTrials.gov, NCT03115437, 11/04/2017) with a biomechanical running analysis at baseline and a 6-month follow-up on running exposure and injury risk, comparing two running shoe prototypes which only differed regarding their cushioning properties. The full trial protocol has been previously published (Malisoux et al., 2017), with some of the methods relevant to this study described below. Reporting of the study followed the CONSORT (Consolidated Standards of Reporting Trials) statement (Moher et al., 2010). The study was approved by the National Ethics Committee for Research (Ref: 201701/02 v1.1). All volunteers received a full description of the protocol and provided written informed consent for participation.

### Participants

Recreational runners were recruited through advertisements in public media and social networks in Luxembourg from September 2017 to January 2018. An a priori sample size calculation estimated that 802 participants were required for the trial (Malisoux et al., 2017). Volunteers in good health, aged 18–65 years, and capable of performing 15 minutes of consecutive running were included in the study. Exclusion criteria were any medical contraindication to perform running activity, prior (<12 months) surgery at the lower limbs or lower back region, use of orthopedic insoles for running or any running-impeding injury over the previous month.

### Intervention

**Figure 1:**
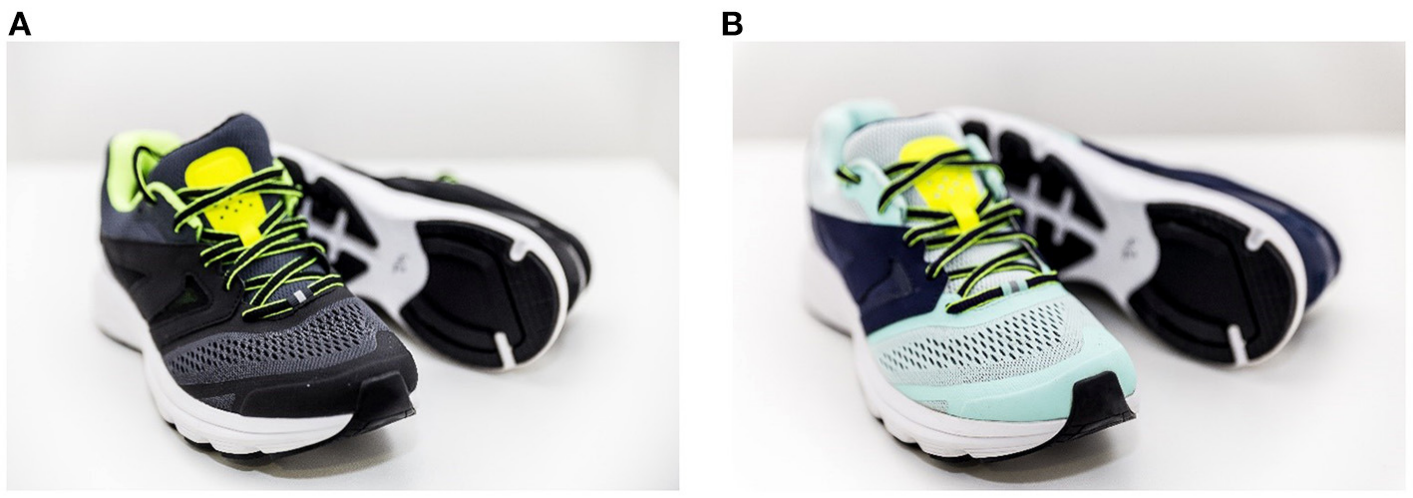
Study shoes for men **(A)** and women **(B)**.

Participants randomly received one of the two running shoe versions specifically designed for the trial and provided by a sport equipment manufacturer (Figure 1). The shoe versions only differed in their cushioning properties, defined by their global stiffness at the heel using an impact test (94.9 ± 5.9 and 61.3 ± 2.7 N/mm in the Hard and Soft versions, respectively), (Malisoux et al., 2020, 2021). Both shoe versions had a heel-to-toe drop of 10 mm and stack height at the heel of 34 mm. Participants were stratified according to sex, known to influence many anthropometric characteristics, and blinded regarding their group allocation. The shoe code was broken after completion of data collection.

### Baseline Evaluations

Prior to their visit to the laboratory, participants filled out an online questionnaire on their running experience and previous injury (past 12 months). The research team measured the participants' height, body mass, fat mass proportion (Tanita SC-240 MA) and leg length (distance between the anterior superior iliac spine and the medial malleolus, in supine position). The running test was performed in the allocated study shoes on a split-belt treadmill instrumented with force plates (M-Gait, MotekForcelink Amsterdam, The Netherlands). The test consisted of three parts: a 3-min warm-up, an 8-min habituation phase at the participant's self-declared preferred running speed, and a 2-min data recording. The full 2-min record was included in the analysis. Participant's body weight was obtained by averaging the vertical signal (Fz) of a 5-s record during quiet stance. GRF data were sampled at 2 kHz.

### Follow-Up Procedures

The participants self-reported all their sports activities, running session characteristics (duration and distance) and any adverse events (injuries, pain and illnesses) during any given session on a dedicated electronic platform throughout the prospective follow-up (Malisoux et al., 2013; Theisen et al., 2014). For each running session reported, mandatory information included, amongst others, the shoe pair used and whether the participant had experienced any pain during the session. Compliance of the participants with the intervention was defined as the percentage of running sessions performed in the study shoes (Nielsen et al., 2020). A running-related injury was defined as a “running-related (training or competition) musculoskeletal pain in the lower limbs that causes a restriction or stoppage of running (distance, speed, duration, or training) for at least seven days or three consecutive scheduled training sessions, or that requires the runner to consult a physician or other health professional” (Yamato et al., 2015). The research team checked all injury data for coherence and contacted the participants by phone or email for a final check of all recordings.

### Signal Processing

**Figure 2:**
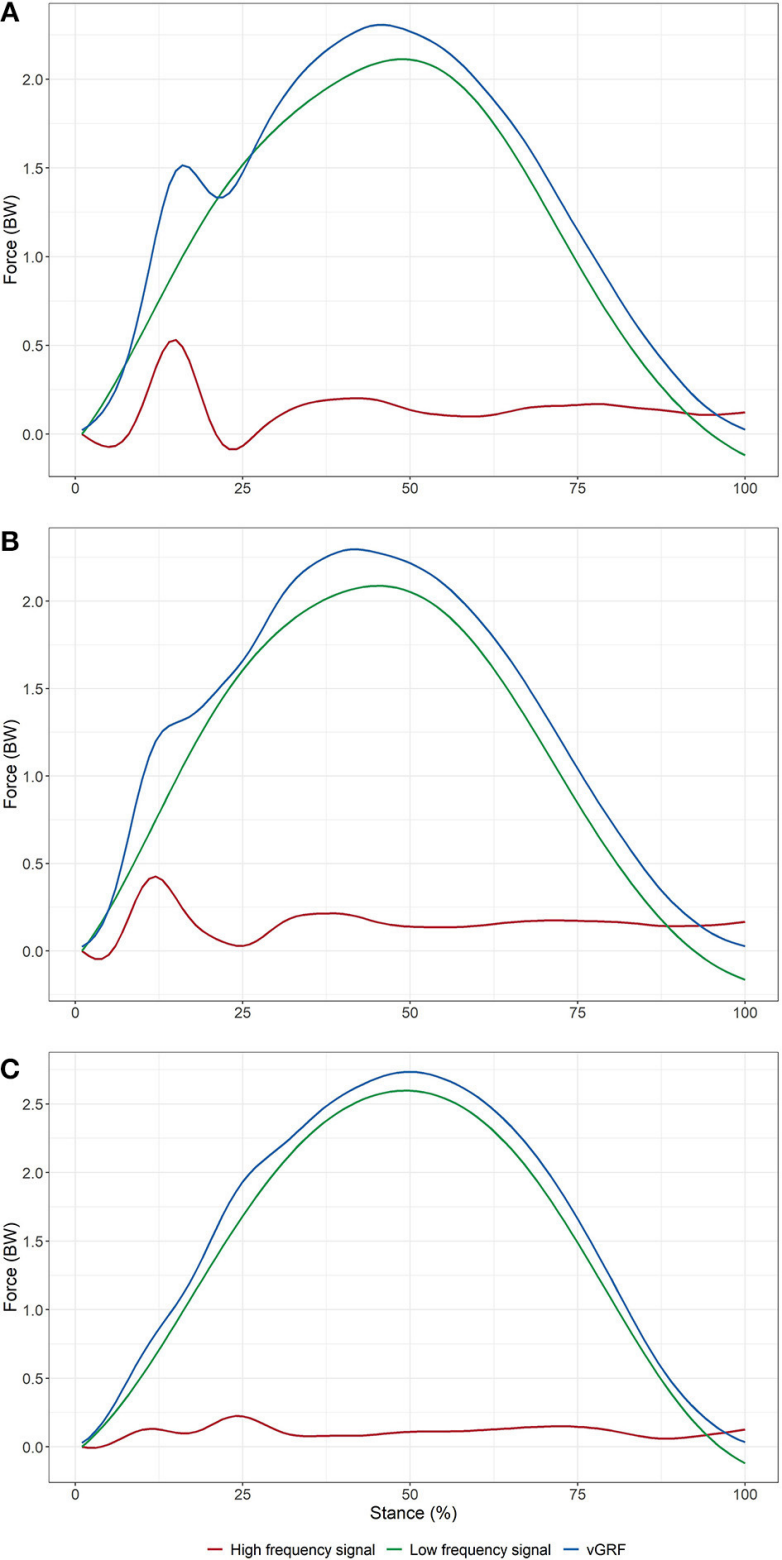
Exemplary ensemble-averaged curves from three participants with 100% **(A)**, 50% **(B)** and 0% **(C)** of their steps with an impact peak identified on the vertical GRF signal, respectively. Blue line is the participant's ensemble-averaged mean of the vertical GRF signal (vGRF), red line and green line are the reconstructed high and low frequency signal, respectively.

A custom program written in Matlab (Matlab R2014a, Math Works, Netherlands) was used to process the GRF data. GRF signal was first filtered using a cut-off frequency of 30 Hz with a bidirectional second order Butterworth low pass filter. Initial contact and toe-off events were identified by Fz exceeding or falling below a 20-N threshold. Frequencies present in the vertical GRF signal in running have been successfully analyzed using the Discrete Fourier Transform. This method is well-understood and commonly used in running and walking (Gruber et al., 2011; Shorten and Mientjes, 2011; Blackmore et al., 2016). While the main disadvantage is the loss of the time resolution of a signal's frequency component, high and low frequency components can be recomposed into the time domain using the inverse Fourier transform to calculate timing features of the two new signals. The Discrete Fourier Transform can be applied to time domain samples of any length and is more accurate than Fast Fourier Transform when applied to non-stationary pulses (Shorten and Mientjes, 2011). We used the Matlab script provided by Blackmore et al. (Blackmore et al., 2016) to decompose the vertical GRF signal for each stance phase into the frequency domain and to separate the high and low frequency components of the signal using 10 Hz as cut-off value (Gruber et al., 2011; Shorten and Mientjes, 2011; Blackmore et al., 2016). In the frequency domain, we computed the signal power of the high frequency component to obtain the weighted mean frequency, the frequency corresponding to the peak signal power, as well as the power sum of the high frequency component (Kiernan et al., 2017). Both high and low frequency components were then recomposed into the time domain using the inverse Fourier transform to form the two new signals (the high and low frequency signals, respectively-Figure 2) and compute the variables of interest (Shorten and Mientjes, 2011; Blackmore et al., 2016). Impact peak force was defined as the maximal value of the high frequency signal, and time to impact peak force was defined as the time between initial contact and impact peak force. Vertical instantaneous loading rate was the maximal slope of the high frequency signal during the same period. Vertical average loading rate was calculated as the average slope in the high frequency signal between 20 and 80% of the period between initial contact and impact peak force (Blackmore et al., 2016). Active peak force was the highest value observed in the low frequency signal, while time to active peak force was defined as the time between initial contact and active peak force.

Force characteristics calculated from the Discrete Fourier Transform were normalized by the participant's body weight. Discrete variables were calculated on each gait cycle of the 2-min record, and then averaged per subject. Data from both limbs were averaged. The GRF data were time-normalized to 101 data points for data visualization.

### Statistical Analysis

Descriptive data for personal, training and biomechanical characteristics are presented as count and percentage for dichotomous variables, and as mean and standard deviation (SD) or as median and interquartile range (IQR), for normally or non-normally distributed continuous variables, respectively. Average running exposure characteristics were computed for each participant over their individual period of observation.

The means of the force characteristics were compared between the two study groups using an analysis of variance (ANOVA), with the participant's preferred running speed as co-variable. The significance level was set at *p* < 0.05. Effect sizes were reported using Cohen's *d* (Cohen, 1988), and were interpreted as negligible (0 to < 0.15), small (0.15 to <0.40), medium (0.40 to <0.75), large (0.75 to <1.10), and very large (≥1.10).

The association between the force characteristics (i.e., predictors) and injury risk was investigated by estimating the sub-distribution hazard using competing-risks regression models, according to Fine and Gray (Fine and Gray, 1999). The primary outcome was the first running-related injury occurred during the 6-month follow-up period. Time at risk was calculated as hours of running between baseline evaluation date and date of injury or censoring (Nielsen et al., 2016). Participants were right-censored if lost to follow-up or at the end of the 6-month observation period. Each force characteristic was categorized. Since no cut-off values have been proposed so far to define populations at higher or lower injury risk with regard to force characteristics, the cut-off values were defined as ±1 SD from the mean, where values outside of that range were considered below or above the reference group, respectively (Jungmalm et al., 2020). The assumption of proportional hazards was evaluated using log-minus-log plots and Schoenfeld's global test. The recommendation for using at least 10 injuries per predictor included in the regression analysis was strictly followed (Peduzzi et al., 1995). First, unadjusted models were used to present the crude estimates of SHR and their 95% CI for each force characteristics separately. Second, each predictor was adjusted for shoe version (the intervention) and previous injury, which is the most common risk factor for running injury (Hulme et al., 2017). All analyses were performed using STATA/SE version 15.

## Results

### Participants

**Figure 3:**
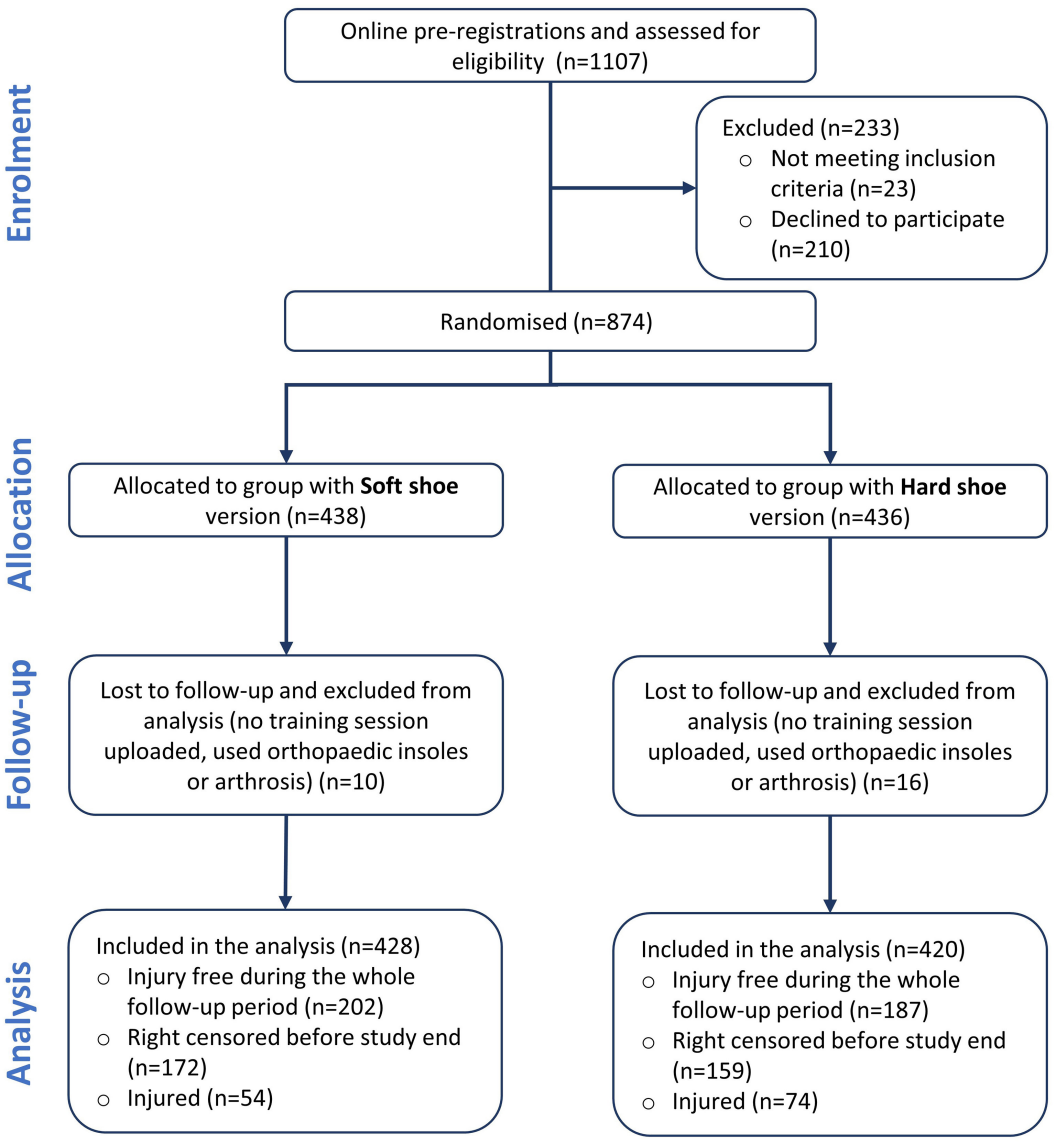
Flow chart of volunteers and study participants.

Out of the 1,107 volunteers who pre-registered for the study on the dedicated electronic platform, 874 recreational runners fulfilled the inclusion criteria, received a pair of running shoes according to the randomized group allocation, and performed the running test in the laboratory. Over the 6-month follow-up period, 26 participants were excluded from the analyses because they did not upload training data (*n* = 19), were diagnosed with arthrosis after inclusion in the study (*n* = 3), reported implausible training data (*n* = 2), used orthopedic insoles (*n* = 1) or had another health issue (*n* = 1). Figure 3 provides an overview of the inclusion and follow-up process.

The characteristics of the 848 participants included in the analysis are presented in Table 1. During the 6-month intervention, they covered 220,014 km (118,864 km and 101,150 km, for the Soft and Hard shoe group, respectively) for a total of 22,521 h of running practice (12,105 and 10,417 h, for the Soft and Hard shoe group, respectively). The participants reported having used the study shoes for more than 97% of the running sessions (85% of the participants never used another pair of running shoes). A detailed report of the compliance with the intervention was published elsewhere (Nielsen et al., 2020).

**Table 1:** Descriptive statistics of the study participants (*n* = 848).

**Variable**	**Unit/qualifier**	**Hard shoe group** **(*n* = 420)**	**Soft shoe group** **(*n* = 428)**
*Personal characteristics*			
Sex	Male	260 (61.9%)	259 (60.5%)
	Female	160 (38.1%)	169 (39.5%)
Age	year	40.5 ± 9.8	40.4 ± 10.2
Body mass	kg	73.3 ± 12.4	73.2 ± 12.8
Running experience^a^	year	6 [0–40]	6 [0–45]
Previous injury	No	711 (83.8%)	711 (83.8%)
	Yes	137 (16.2%)	137 (16.2%)
*Running exposure*			
Weekly running distance^a^	km.week^−1^	10.5 [6.2–17.9]	11.3 [6.3–18.7]
Weekly running volume^a^	min.week^−1^	65 [41–109]	69 [41–115]
Mean running speed	km.h^−1^	9.6 ± 1.5	9.7 ± 1.4
Running frequency^a^	sessions.week^−1^	1.3 [1.0–2.0]	1.4 [1.0–2.0]

*^a^Non-normally distributed variable presented as median [interquartile range]*.

### Injuries

During the intervention, 220 participants reported at least one injury. For 128 of these participants, the first injury met the definition of a running-related injury (as described in the methods), and for 92 of them, the injury was defined as a competing injury (e.g., fall in the stairs at home, knee sprain during skiing, etc.). The overall running-related injury incidence was 5.7 injuries per 1,000 h of running (95% CI: 4.8 to 6.8).

The ankle (26.5%), knee (21.9%), lower leg (18.8%), and foot (15.6%) were the most frequent running-related injury locations. Almost half of the running-related injuries (48.4%) were tendinitis, and 19.5% of the running-related injuries were muscle injuries. About 93% of the running-related injuries were reported in the context of training, while only 14% were acute injuries. Additional information on the running-related injuries were previously published (Malisoux et al., 2020).

### Effect of Shoe Cushioning on Force Characteristics

**Figure 4:**
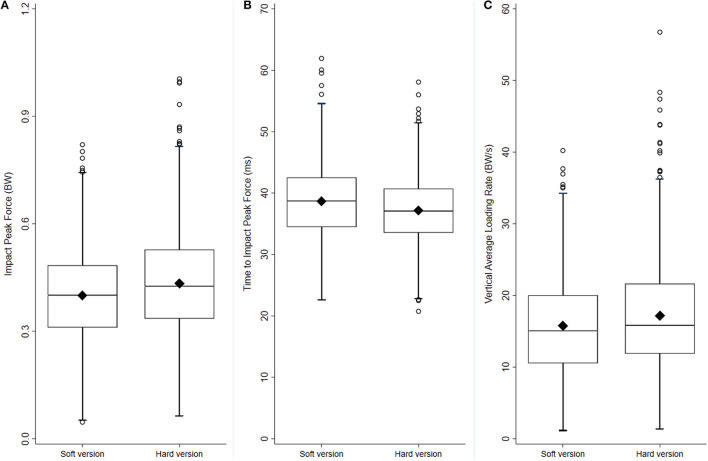
Box plots for impact peak force [BW, *p* < 0.001, **(A)**], time to impact peak force [ms, *p* < 0.001, **(B)**] and vertical average loading rate [BW.s^−1^, *p* = 0.006, panel **(C)**] of the high frequency signal, in the two experimental groups (*n* = 428 and 420 for the Soft and Hard shoe group, respectively). The lower and upper box boundaries indicate the 25th and 75th percentiles, respectively, the middle line inside the box represents the median, and the filled diamond is the mean. Whiskers below and above the boxes represent the 25th percentile−1.5*interquartile range (IQR) and 75th percentile +1.5*IQR, respectively. Empty circles are data falling outside the range of the whiskers.

Participants' preferred running speed at baseline was 9.8 ± 1.5 and 9.9 ± 1.5 km.h^−1^ in the Soft and Hard shoe group, respectively. On average, 326 ± 19 and 325 ± 19 steps were analyzed per participant in the Soft and Hard shoe group, respectively. Descriptive and inferential statistics for the force characteristics are presented in Table 2. Regarding the high frequency force component, the analysis revealed significantly lower impact peak force (95% CI: 0.01 to 0.05 BW; small effect, *d* = 0.23) and longer time to impact peak force (95% CI: 0.7 to 2.2 ms; small effect, *d* = 0.25) in the Soft shoe group compared to the Hard shoe group (Figure 4). Vertical average loading rate was significantly lower in the Soft shoe group (95% CI: 0.4 to 2.2 BW.s^−1^; small effect, *d* = 0.18), but no difference was observed for vertical instantaneous loading rate. The Soft shoe group also showed lower weighted mean frequency (95% CI: 0.6 to 0.8 Hz; medium effect, *d* = 0.67) and frequency of the peak signal power (95% CI: 0.2 to 0.4 Hz; small effect, *d* = 0.32). No difference was observed in the high frequency signal power sum between shoe groups.

**Table 2:** Effect of shoe version on force characteristics of the high and low frequency signals (*n* = 848).

**Variable**	**Unit**	**Hard shoe group (*n* = 420)**	**Soft shoe group (*n* = 428)**	**Mean absolute difference**	***p*-value**
*High frequency signal*					
Impact peak force	BW	0.43 ± 0.16	0.40 ± 0.14	0.03	<0.001*
Time to impact peak force	ms	37 ± 6	39 ± 6	1.5	<0.001*
Vertical instant. loading rate	BW.s^−1^	32.2 ± 12.5	31.7 ± 10.4	0.3	0.643
Vertical average loading rate	BW.s^−1^	17.2 ± 8.4	15.7 ± 7.4	1.3	0.006*
Weighted mean frequency	Hz	16.5 ± 1.2	15.8 ± 1.0	0.7	<0.001*
Frequency of peak signal power	Hz	12.4 ± 1.0	12.1 ± 0.7	0.3	<0.001*
Power sum^a^	BW^2^.Hz^−1^	4.11 ± 2.50	3.98 ± 2.13	0.13	0.539
*Low frequency signal*					
Active peak force	BW	2.06 ± 0.21	2.05 ± 0.20	0.01	0.565
Time to active peak force	ms	131 ± 19	129 ± 20	2.2	0.044*

Preferred running speed is cofactor; ^a^Divided by 10^3^ for readability; BW, bodyweight; *p-value < 0.05.

**Figure 5:**
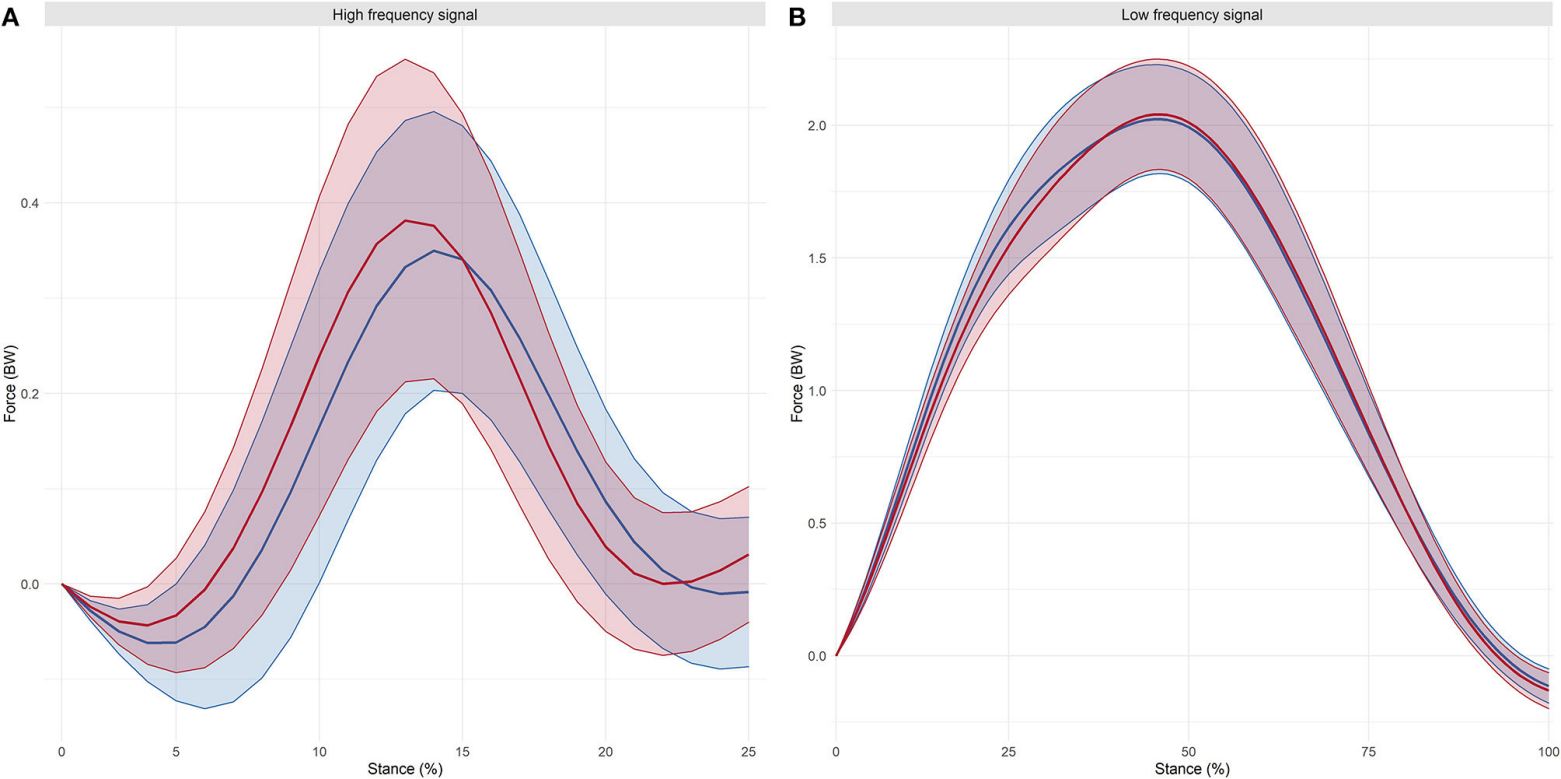
Ensemble-averaged curves of the high **(A)** and low **(B)** frequency signals in the two experimental groups (Red: Hard shoe group; Blue: Soft shoe group). Thick line is the shoe group's ensemble-average mean, and thin lines are 1 SD below and above the mean.

Analysis of the low frequency signal showed no difference between the shoe groups for active peak force, while time to active peak force was shorter in the Soft shoe group (95% CI: −4 to 0 ms; negligible effect, *d* = 0.09). Ensemble-averaged curves of high and low frequency signals in the two experimental groups are presented in Figure 5.

### Association Between Force Characteristics and Injury Risk

**Figure 6:**
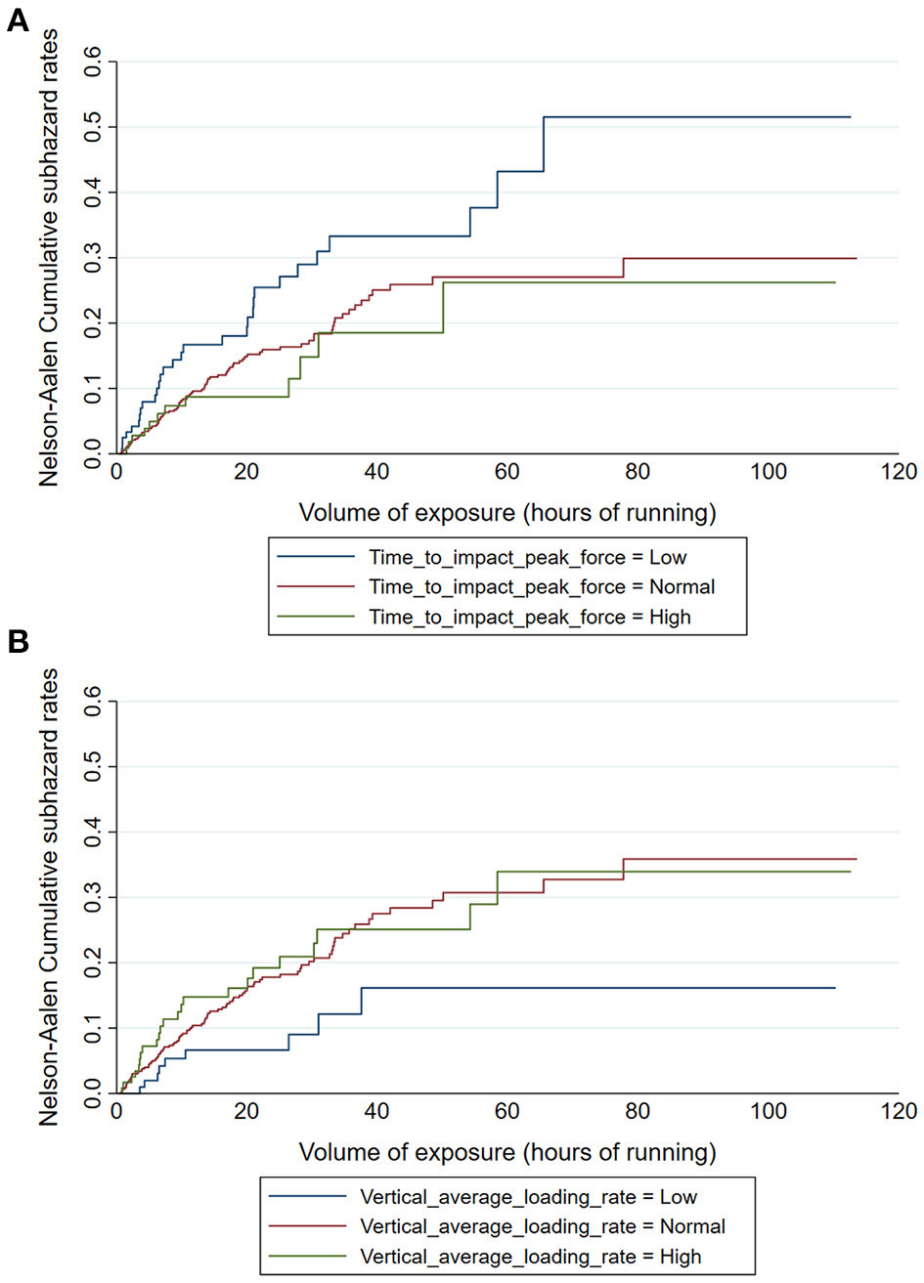
Cumulative incidence functions for running-related injury according to time to impact peak force [ms, **(A)**] and vertical average loading rate [BW.s^−1^, **(B)**] of the high frequency vertical GRF signal.

Table 3 presents the crude estimates of the SHR for each force characteristic of the high and low frequency signals. Participants with low time to impact peak force (i.e., those in the −1 SD group) had a greater risk of running-related injury than the reference group (Figure 6A), and those with low vertical average loading rate had a lower running-related injury risk (Figure 6B). No other association with running-related injury was observed among the force characteristics in the low and high frequency signal.

**Table 3:** Unadjusted competing risk regression models for the association between force characteristics of the high and low frequency signals and running-related injury risk (*n* = 848).

**Variable**	**Unit**	**Ref**.	**1 SD below ref**.		**1 SD above ref**.	
		**Injuries (part.)**	**Injuries (part.)**	**SHR (95% CI)**	**Injuries (part.)**	**SHR (95% CI)**
				***p*-value**		***p*-value**
*High frequency signal*						
Impact peak force	BW	96 (603)	13 (126)	0.64 (0.36–1.13) *0.127*	19 (119)	0.92 (0.56–1.51) *0.728*
Time to impact peak force	ms	86 (609)	30 (125)	1.65 (1.09–2.51) 0.018*	12 (114)	0.80 (0.44–1.46) *0.461*
Vertical instant. loading rate	BW.s^−1^	99 (595)	12 (135)	0.57 (0.31–1.03) *0.061*	17 (118)	0.75 (0.45–1.25) *0.263*
Vertical average loading rate	BW.s^−1^	96 (608)	9 (118)	0.49 (0.25–0.97) 0.040*	23 (122)	1.11 (0.70–1.77) *0.647*
Weighted mean frequency	Hz	96 (622)	11 (115)	0.65 (0.34–1.22) *0.178*	21 (111)	1.21 (0.76–1.92) *0.434*
Freq. of peak signal power	Hz	101 (689)	3 (32)	0.77 (0.25–2.32) *0.640*	24 (127)	1.25 (0.80–1.94) *0.323*
Power sum^a^	BW^2^.Hz^−1^	95 (598)	17 (127)	0.86 (0.51–1.44) *0.572*	16 (123)	0.75 (0.44–1.27) *0.280*
*Low frequency signal*						
Active peak force	BW	620 (101)	116 (11)	0.54 (0.29–1.01) 0.051	112 (16)	0.79 (0.46–1.35) 0.388
Time to active peak force	ms	590 (89)	145 (28)	1.12 (0.74–1.71) 0.595	113 (11)	0.69 (0.37–1.29) 0.245

^a^Divided by 10^3^ for readability; Part., Participants; SD, Standard Deviation; SHR, Sub hazard rate ratio; BW, bodyweight; CI, Confidence Interval; *p-value < 0.05.

Table 4 shows the estimates of the SHR for each force characteristic of the high and low frequency signals after adjustment for previous injury and shoe version. The group of participants with low time to impact peak force was still significantly associated with greater injury risk compared to the reference group, and those with low vertical average loading rate had lower injury risk. Furthermore, runners with low vertical instantaneous loading rate had lower risk of running-related injury than the reference group in the adjusted model.

**Table 4:** Adjusted competing risk regression models for the association between force characteristics of the high and low frequency signals and running-related injury risk (*n* = 848).

**Variable**	**Unit**	**Ref**.	**1 SD below ref**.		**1 SD above ref**.	
		**Injuries (part.)**	**Injuries (part.)**	**SHR (95% CI)**	**Injuries (part.)**	**SHR (95% CI)**
				***p*-value**		***p*-value**
*High frequency signal*						
Impact peak force	BW	96 (603)	13 (126)	0.63 (0.36–1.12) *0.117*	19 (119)	0.83 (0.50–1.37) *0.464*
Time to impact peak force	ms	86 (609)	30 (125)	1.60 (1.05–2.43) 0.028*	12 (114)	0.83 (0.45–1.51) *0.534*
Vertical instant. loading rate	BW.s^−1^	99 (595)	12 (135)	0.55 (0.30–0.99) 0.050*	17 (118)	0.71 (0.42–1.20) *0.203*
Vertical average loading rate	BW.s^−1^	96 (608)	9 (118)	0.49 (0.25–0.97) 0.040*	23 (122)	1.03 (0.65–1.66) *0.887*
Weighted mean frequency	Hz	96 (622)	11 (115)	0.695 (0.37–1.30) *0.249*	21 (111)	1.05 (0.65–1.71) *0.846*
Freq. of peak signal power	Hz	101 (689)	3 (32)	0.84 (0.28–2.51) *0.762*	24 (127)	1.18 (0.76–1.85) *0.461*
Power sum^a^	BW^2^.Hz^−1^	95 (598)	17 (127)	0.83 (0.50–1.40) *0.492*	16 (123)	0.70 (0.41–1.19) *0.189*
*Low frequency signal*						
Active peak force	BW	620 (101)	116 (11)	0.54 (0.29–1.01) 0.051	112 (16)	0.77 (0.45–1.33) 0.348
Time to active peak force	ms	590 (89)	145 (28)	1.18 (0.77–1.80) 0.450	113 (11)	0.68 (0.36–1.28) 0.230

^a^Divided by 10^3^ for readability; Adjusted for previous injury and shoe version; Part., Participants; SD, Standard Deviation; SHR, Sub hazard rate ratio; BW, bodyweight; CI, Confidence Interval; *p-value < 0.05.

**Figure 7:**
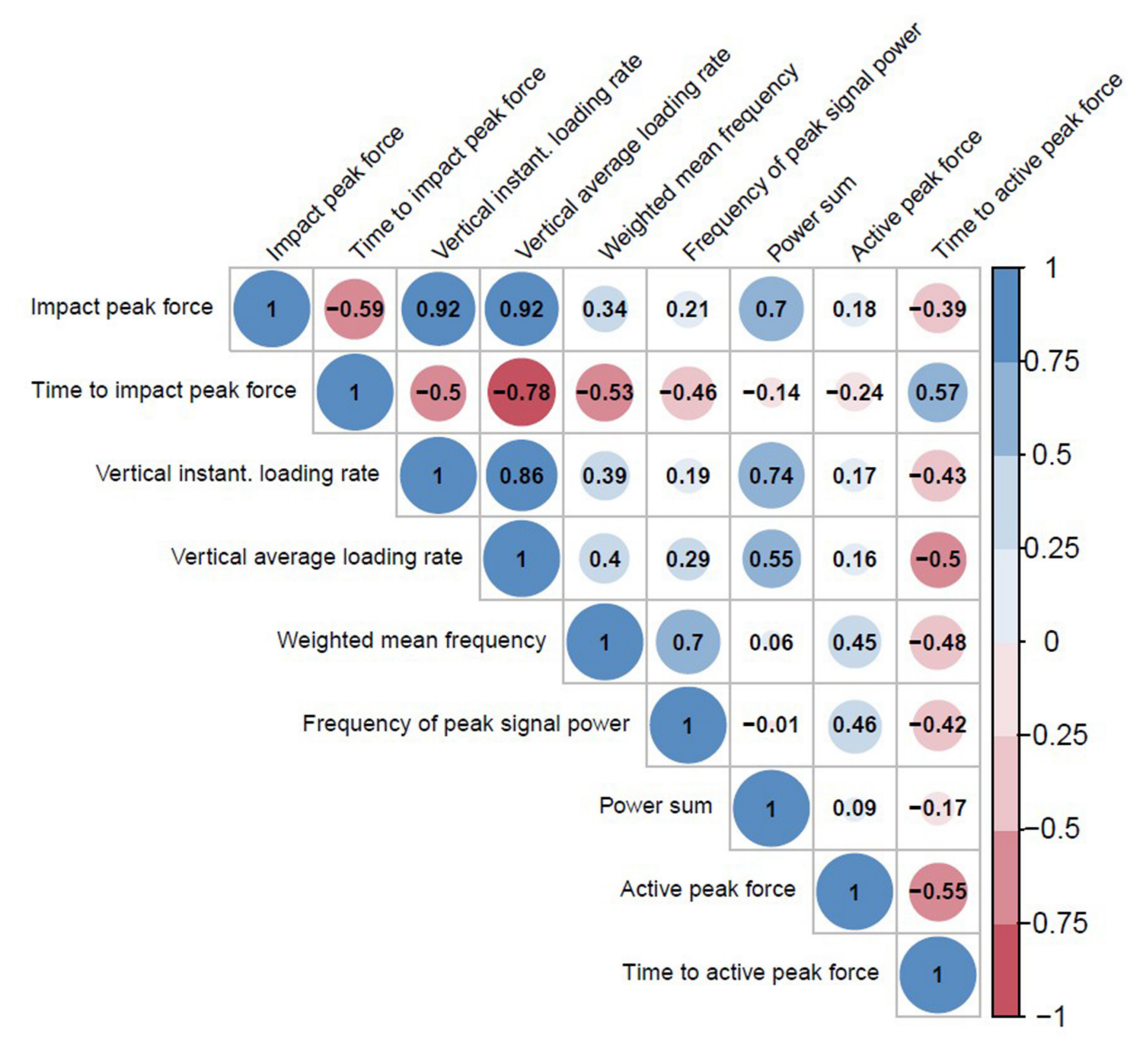
Correlation matrix between the force characteristics. The size of the circle is proportional to the correlation. Blue colors and red colors indicate positive and negative correlations, respectively.

The correlation matrix (Figure 7) revealed that each force characteristic was correlated with many others, which prevents from building further multivariable regression models by including several of these force characteristics in the same model.

## Discussion

### Main Findings

The main purpose of this study was to investigate whether the protective effect of the Soft shoe version observed previously in recreational runners (Malisoux et al., 2020) could be explained by differences in the decomposed impact force characteristics using frequency-domain analyses. Therefore, the first aim of the study was to investigate the effect of shoe cushioning on the time, magnitude and frequency characteristics of peak forces using frequency-domain analyses in a cross-sectional approach comparing the two study groups at baseline (i.e., participants who received the Hard and Soft shoe version, respectively). We hypothesized that using the Soft shoe version would be associated with a lower impact peak force, a longer time to impact peak force, and lower vertical average loading rate and vertical instantaneous loading rate of the high-frequency signal (Shorten and Mientjes, 2011), as well as with lower frequency of the peak signal power, weighted mean frequency and power sum. In line with these hypotheses, we observed a lower impact peak force, a longer time to impact peak force and a lower vertical average loading rate in the Soft shoe group compared to the Hard shoe group. Furthermore, the frequency-domain analysis also revealed lower weighted mean frequency and frequency of the peak signal power in the Soft shoe group. No difference was observed for vertical instantaneous loading rate and power sum between the experimental groups. Altogether, the observations are congruent and support our hypothesis that the Soft shoe version decreased the magnitude and frequency characteristics of impact forces (and thus, the timing of occurrence as well) when compared to the Hard shoe version. Although the effect sizes were small to medium, these differences may be clinically relevant given the thousands of steps cumulated over every single running session. Consequently, careful investigation into the role of impact force characteristics in the development of running-related injury is needed.

The secondary objective of this study was to investigate which of these force characteristics measured at baseline are prospectively associated with the risk of running-related injury in the same cohort of runners. We hypothesized that a lower impact peak force, a longer time to impact peak force, and lower vertical instantaneous loading rate and/or vertical average loading rate of the high-frequency signal would be associated with lower injury risk. We also expected that lower frequency of the peak signal power, weighted mean frequency, as well as power sum would be associated with lower injury risk. The crude analysis revealed that the participants with low vertical average loading rate had lower injury risk than the reference group, which supports our hypothesis. Furthermore, those with short time to impact peak force had greater injury risk. After adjustment for previous injury and shoe version, the participants with low vertical average loading rate as well as those with low vertical instantaneous loading rate had lower risk of running-related injury than the reference group. Short time to impact peak force remained associated with greater injury risk. These observations are consistent and partially support our hypothesis. This is the first study showing the association between loading rate and running-related injury risk using data from a large prospective cohort study in combination with frequency-domain analyses of ground impact forces. Our findings also suggest that Discrete Fourier Transform is appropriate to investigate the effectiveness of preventive measures aiming at decreasing impact forces on injury risk.

### Cushioning and Impact Forces

To date, most studies investigating the effect of shoe cushioning on impact forces used the first peak of the vertical GRF (Fz1) as a measure of impact intensity and an indicator of running shoe impact attenuation (Chan et al., 2018b; Kulmala et al., 2018; Pollard et al., 2018; Malisoux et al., 2021). Globally, these studies reported greater Fz1 and vertical loading rate in shoes with greater cushioning compared to the control condition. Although this observation is consistent, it is counterintuitive, as cushioning systems aim to attenuate this first peak. Furthermore, results from *in vitro* mechanical tests of running soles meet expectations based on impact attenuation theory (Shorten and Mientjes, 2011; Theisen et al., 2014). This discrepancy between *in vitro* mechanical tests and *in vivo* measurements of Fz1 has been termed “the shoe cushioning paradox” (Kulmala et al., 2018) or “the impact peak anomaly” (Shorten and Mientjes, 2011). Some authors have suggested that a mechanistic explanation for this phenomenon may lie in neuro-muscular adaptations (Kulmala et al., 2018). Others have demonstrated that the apparent increased Fz1 in softer shoes is actually the consequence of a greater contribution of the low frequency signal added to the delayed (and attenuated) high frequency impact peak force (Shorten and Mientjes, 2011). The present study fully aligns with this explanation, as we observed (1) an attenuated impact peak force of the high frequency signal, (2) a delayed timing of occurrence of that impact peak force, and (3) lower vertical (average) loading rate of the high frequency signal in the runners using the Soft shoe version compared to those using the Hard shoes. Furthermore, the frequency at which the maximal power of the high frequency signal was observed, and the weighted mean frequency of the high frequency signal were also lower in the Soft shoes. The latter finding is consistent with previous observations (Shorten and Mientjes, 2011). As expected, no difference was observed between the experimental groups in the peak force of the low frequency signal. Nevertheless, we observed a negligible shorter time to active peak force (1.7%, 95% CI: −4 to 0 ms) in the Soft shoe group, while no difference in ground contact time was observed between the study groups, as reported in a previous article (Malisoux et al., 2021).

Altogether, our results confirm that greater shoe cushioning is associated with reduced high frequency (impact) loads and delayed timing of occurrence. They also support previous contributions suggesting that impact force characteristics measured on the global vertical GRF signal are not appropriate markers of impact intensity, and consequently running shoe impact attenuation (Shorten and Mientjes, 2011; Malisoux et al., 2021). A relevant and meaningful marker of impact intensity can only be defined using appropriate frequency-domain analyses such as the Discrete Fourier Transform (Gruber et al., 2011; Shorten and Mientjes, 2011; Blackmore et al., 2016). Furthermore, the present findings also provide a first piece of evidence that the protective effect of greater cushioning observed in our previous trial (Malisoux et al., 2020) may be related to the reduction of impact forces of the high frequency signal.

### Impact Forces and Injury Risk

The role of impact force characteristics in the development of running-related injury has received much interest from the scientific community over the last decades. Some cross-sectional studies indicated that vertical loading rate is higher in patients with a history of stress fracture (van der Worp et al., 2016) or overuse injury (Hreljac et al., 2000) compared to healthy subjects. However, few studies have provided prospective evidence that impact force characteristics are related to injury risk (Ceyssens et al., 2019). Considering the design of studies conducted so far, their limited sample size, as well as the different populations investigated and the main outcomes (i.e., injury definition), current evidence relating impact force characteristics and injury risk is sparse and inconclusive (Nigg et al., 2015; Theisen et al., 2016; Ceyssens et al., 2019). The present study is the largest, and one of the few prospective studies on the association between force characteristics and injury risk in recreational runners so far. Furthermore, despite the increasing evidence on the inaccuracy of Fz1 as a marker of impact intensity, this is the first study to apply frequency-domain analyses to the vertical GRF signal and to focus on high frequency loads as injury risk factors using a prospective approach. The main findings from the adjusted model (controlled for previous injury and shoe version) are consistent and show that runners with lower vertical (average or instantaneous) loading rate of the high frequency signal have lower injury risk, and those with earlier occurrence of impact peak force have greater injury risk when compared to the reference group. These results also suggest that an intervention aiming at lowering vertical loading rate and delaying impact peak force occurrence may potentially reduce injury risk in running. This is a second piece of evidence that the protective effect of greater cushioning observed in our previous trial may be related to the attenuation of impact force of the high frequency signal.

Another methodological concern is that the relationship between impact force characteristics and injury risk might not be linear, but U-shaped (Bahr and Holme, 2003; Jungmalm et al., 2020). In such a case, impact force characteristics cannot be used as continuous variables in a regression model but they should be categorized using cut-off points. Ideally, these cut-off points should correspond to previously identified normative values or clinically meaningful thresholds. Given the absence of such established reference values, we used 1 SD below and above the cohort mean to create the reference group and the groups of runners with low and high values, respectively, for each force characteristic. Future research should confirm the shape of the relationship between different biomechanical variables and injury risk, and if possible, provide reference values that identify groups of runners at greater injury risk.

### Clinical Implications

Running is characterized by the repetition of high impact forces applied to the musculoskeletal system. The cumulative effect resulting from tens of thousands of steps over time can progressively lead to injury if the cumulative load applied to a specific structure exceeds the structure-specific load capacity (Bertelsen et al., 2017). Therefore, paradigms leading to a decrease of impact forces, such as shoe cushioning, may potentially reduce injury risk. In a randomized trial including 848 recreational runners followed up for 6 months, we previously found that the participants having received the Soft shoe version had a lower injury risk compared to those having received the Hard version (Malisoux et al., 2020). The present study is a secondary analysis of the data from the same trial and aims to provide answers to three main practical questions. (1) Does shoe cushioning attenuate impact forces in running? (2) Do impact force characteristics influence injury risk in recreational runners? (3) According to the answer to the first two questions, is a decrease in impact forces a plausible explanation to the protective effect provided by the Soft shoe version observed in our previous trial? Of course, this third question is indirectly addressed and remains partially speculative. First, we demonstrated that shoe cushioning attenuates impact force characteristics of the high frequency signal. Thus, cushioning appears to be of particular importance for runners to prevent the occurrence of some types of injury or recovering from an injury (e.g., stress fracture), provided that there is evidence for a role of impact force in the underlying mechanism of that injury. Through the second objective of this study, we revealed that some impact force characteristics, namely vertical loading rate and time to impact peak force of the high frequency signal (both being negatively correlated–see Figure 7), are associated with injury risk. This indicates that paradigms aiming at reducing vertical loading rate such as greater cushioning or gait retraining (Chan et al., 2018a) may be efficient to prevent running-related injury. Future intervention studies should be designed to test the effect of a reduced vertical loading rate of the high frequency component on injury risk in different populations of runners and define thresholds for the identification of runners at risk. Furthermore, more research should investigate the injury types where impact force characteristics play a role in the underlying mechanism. Altogether, our findings suggest that a decrease in impact forces of the high frequency signal is a plausible explanation to the protective effect provided by the Soft shoe version.

### Strengths and Limitations

To the best of our knowledge, this is the first study investigating the association between shoe cushioning, impact force and the risk of running-related injury in a very large cohort of recreational runners over 6 months using frequency-domain analyses of ground impact forces. Additionally, many steps were analyzed per participant (326 ± 19 steps) in the allocated shoe conditions, which increases the representativeness of the measurements. The large sample size allowed the identification of small differences in the mean effect between shoe versions that may be clinically relevant given the thousands of steps cumulated during each running session.

The present study also has some limitations. The use of a rather inclusive definition for running-related injury may have influenced the findings as some of the force characteristics may be related to the development of some types of injury, but not to all of them. Some features of the study design also limit the generalization of the findings. More competitive runners might be underrepresented in this study, because of a lower readiness to comply with the study requirements (i.e., the use of the study shoes was mandatory for all running sessions), and the influence of shoe cushioning on impact forces might be greater at higher speeds. However, these competitive runners might not represent the main proportion of recreational runners. The vertical ground reaction force signal was recorded in a laboratory setting. Some other factors such as running surface, slope and training plan, obviously influence GRF characteristics and were not taken into account here. The test was performed on an instrumented treadmill. Given the heterogeneity of our study population (see Table 1), there may be large individual variability in how runners change their biomechanics on a treadmill compared to over ground running. Finally, the study includes a single baseline measurement at a fixed running speed, which is then assumed to represent the runner's technique throughout the follow-up period. Future studies should include the collection of force data in the runners' natural environment through the whole observation period to quantify these impact forces in “real world conditions,” describe their variability, and take the time-varying nature of these factors into account (Nielsen et al., 2016) when investigating associations with the risk of sustaining specific injury types.

## Conclusions

The present study revealed that recreational runners using the Soft shoes displayed lower impact force, longer time to peak, and lower loading rate of the high frequency signal compared to those using the Hard shoe version. Consistently, frequency-related metrics, such as mean frequency of the high frequency signal and frequency at which peak power of the high frequency signal is observed, were both lower in runners using the Soft shoe version. This effect of shoe cushioning on impact forces may explain the protective effect of the Soft shoe version previously observed. The findings from the prospective analysis are in line with this explanation, as runners with a lower loading rate in the high frequency component had lower injury risk, and those with early occurrence of impact peak had greater injury risk. While previous work has questioned the relevance of vertical GRF metrics as measures of impact force, and as indicators of running shoe impact attenuation, musculoskeletal loading and injury risk, the present study demonstrated that clinically relevant impact force characteristics can be obtained using frequency-domain analyses.

## Data Availability Statement

Data are available upon reasonable request to LM. De-identified participant data might be available after the consent of all authors, the business development office of the Luxembourg Institute of Health and Decathlon. Requests to access these datasets should be directed to Laurent Malisoux, laurent.malisoux@lih.lu.

## Ethics Statement

The study involved human participants and was reviewed and approved by Comité National d'Ethique de Recherche Luxembourg. The patients/participants provided their written informed consent to participate in this study.

## Author Contributions

LM, PG, AB, ND, JC, and DT contributed sufficiently to the manuscript to justify authorship. LM, ND, and DT conceptualized the project and defined the methodology. LM and PG collected the data. PG adapted the Custom MATLAB programs. LM, PG, and AB conducted the analysis. All authors were involved in the interpretation of the results. LM drafted the original manuscript, all other authors provided significant feedback, and comments in refining the final manuscript. All authors approved the final manuscript. All authors agree to be accountable for the content of the work.

## Funding

This study was co-funded by Decathlon, Villeneuve d'Ascq, France and by the Ministry of Higher Education and Research, Luxembourg.

## Conflict of Interest

ND is employed at Decathlon. Decathlon also provided the standard running shoes. A research partnership agreement was signed between Decathlon and the LIH. The remaining authors declare that the research was conducted in the absence of any commercial or financial relationships that could be construed as a potential conflict of interest.

## Publisher's Note

All claims expressed in this article are solely those of the authors and do not necessarily represent those of their affiliated organizations, or those of the publisher, the editors and the reviewers. Any product that may be evaluated in this article, or claim that may be made by its manufacturer, is not guaranteed or endorsed by the publisher.
